# Enhancing educational Q&A systems using a Chaotic Fuzzy Logic-Augmented large language model

**DOI:** 10.3389/frai.2024.1404940

**Published:** 2024-08-08

**Authors:** Haoyuan Chen, Nuobei Shi, Ling Chen, Raymond Lee

**Affiliations:** ^1^Guangdong Provincial Key Laboratory of Interdisciplinary Research and Application for Data Science, Beijing Normal University-Hong Kong Baptist University United International College, Zhuhai, China; ^2^Department of Computer Science, Faculty of Science, Hong Kong Baptist University, Hong Kong, China; ^3^School of Applied Science and Civil Engineering, Beijing Institute of Technology, Zhuhai, China

**Keywords:** education, large language model, Lee Oscillator, Fuzzy Logic, AI-based QA system

## Abstract

**Introduction:**

Online question-and-answer (Q&A) platforms are frequently replete with extensive human resource support. This study proposes a novel methodology of a customized large language model (LLM) called Chaotic LLM-based Educational Q&A System (CHAQS) to navigate the complexities associated with intelligent Q&A systems for the educational sector.

**Methods:**

It uses an expansive dataset comprising over 383,000 educational data pairs, an intricate fine-tuning process encompassing p-tuning v2, low-rank adaptation (LRA), and strategies for parameter freezing at an open-source large language model ChatGLM as a baseline model. In addition, Fuzzy Logic is implemented to regulate parameters and the system's adaptability with the Lee Oscillator to refine the model's response variability and precision.

**Results:**

Experiment results showed a 5.12% improvement in precision score, an 11% increase in recall metric, and an 8% improvement in the F1 score as compared to other models.

**Discussion:**

These results suggest that the CHAQS methodology significantly enhances the performance of educational Q&A systems, demonstrating the effectiveness of combining advanced tuning techniques and fuzzy logic for improved model precision and adaptability.

## Introduction

Contents depend on domain specialists to annotate data and establish a robust reference framework for inquiries and answers in classical education. Question-and-answer (Q&A) online platforms, such as Piazza or EdStem, effectively provide answers to students' queries related to course contents, assignments, and administrative procedures (Thinnyun et al., 2021). However, the volume of queries from students at the end of each semester has generated extensive human resource support, in particular to the computer science discipline, where enrollment rates have experienced an unprecedented surge (Thinnyun et al., 2021). In 2015, Jill Watson AI by GeorgiaTech signified an ontology framework with a Q&A pair database to alleviate the loads associated with manual question–answering processes (Goel et al., 2021), but it is restricted to logistical or syllabus-related nature queries involving substantial maintenance and development resources.

Large language models (LLMs), such as ChatGPT (Goel et al., 2021), ChatGLM (Du et al., 2022), and LLaMA2-Chat (Touvron et al., 2023), have exemplified intelligent education to deliver personalized and timely responses in recent years but lack reliability in the educational context. Hence, this study uses a novel methodology of a customized LLM called Chaotic LLM-based Educational Q&A System (CHAQS) by using a vast corpus of educational data to fine-tune an open-source LLM model, ChatGLM3-6b (Du et al., 2022). Meanwhile, Fuzzy Logic with a chaotic Lee Oscillator (Lee, 2004, 2020) dynamically modulates answer generation parameters of the system, such as *temperature* and *top_p*. It uses its dynamic inherent properties enabling adjustment mechanisms to not only generate nuanced and contextually relevant answers within the educational domain but also diverse inquiries and delivering response flexibility. Experiment results showed that it achieved satisfactory performance in Bidirectional Encoder Representations from Transformers (BERT) F1, precision, and recall score evaluation metrics.

The contributions of this study are as follows:

- Build a specialized educational domain LLM for answering questions.
- Use Fuzzy Logic to dynamically adjust output parameters of the model, such as top_p and temperature.
- Propose a Q&A system combining Fuzzy Logic with Lee Oscillator for response flexibility.
- Compare the benchmark model to indicate significant improvements in F1, precision, and recall metrics.

## Related work

### Large language models

LLMs, such as ChatGPT (Goel et al., 2021), ChatGLM (Du et al., 2022), and LLaMA2-Chat (Touvron et al., 2023), signified transformation across various industries enhancing user experiences through many applications but are restricted to specialized domains. These include as follows: OceanGPT focuses on oceanography to leverage vast datasets for research and conservation efforts on marine ecosystems (Bi et al., 2023) and Chatlaw navigates the intricacies of legal language, statutes, and case laws for legal professionals (Cui et al., 2023). HuatuoGPT focuses on medical diagnostics and patient care to provide insights and understanding of complex medical conditions (Zhang et al., 2023). FinGPT analyzes market trends, financial reporting, and investment strategies for finance sectors (Deng and Yu, 2023).

Additionally, ArtGPT interprets artistic contents to facilitate creative processes, providing insights into the history and theory to correlate with technology and creative arts (Yuan et al., 2023). Furthermore, a MathGPT solves complex mathematical problems with interpretations (Rane, 2023).

Each of these models exemplifies the concerted effort to modify LLM capabilities to specific domains, navigating the inherent complexities of the applicability of diverse fields and artificial intelligence.

It is noteworthy that the effects of LLMs on educational domains have shifted to interactive and personalized learning environments, which highlighted the profound implications of chat-based Q&A systems on educational engagement and efficacy (Deng and Yu, 2023). Other educational chatbots, such as Ada (Xie and Pentina, 2022) and the Replika AI platform (Xie and Pentina, 2022), further exemplify LLMs' adaptabilities for academic support and emotional engagement with students. Furthermore, fine-tuning of LLMs for nuanced teacher responses in BEA 2023 Shared Task underscores the continuous efforts on model refinement (Zhao et al., 2020; Dan et al., 2023).

Fine-tuning techniques, such as adapter tuning to pattern-exploiting training (PET; Schick and Schütze, 2021), prefix and prompt tuning, P-tuning (Liu et al., 2022b), P-tuning V2 (Liu et al., 2022a), and low-rank adaptation (LoRA; Hu et al., 2022), have improved model customization with minimal adjustments to the underlying parameters enhancing the adaptability and efficiency of LLMs specific domains.

### Lee Oscillator

Lee Oscillator (Lee, 2004) is a chaotic neural oscillator that simulates the dynamic behavior of neurons and information processing. It exhibits better gradual transitions, shifting from chaotic to non-chaotic states smoothly as compared to traditional neural oscillators. The calculation formula is as follows:


(1) $$\begin{array}{r} u\left(t+1\right)=f\left[a_{1}u\left(t\right)+a_{2}v\left(t\right)+I\left(t\right)-\theta_{u}\right] \end{array}$$

(2) $$\begin{array}{r} v\left(t+1\right)=f\left[b_{1}u\left(t\right)-b_{2}v\left(t\right)-\theta_{v}\right] \end{array}$$

(3) $$\begin{array}{r} S\left(t+1\right)=f\left[I\left(t\right)\right] \end{array}$$

(4) $$\begin{array}{r} z\left(t\right)=\left[u\left(t\right)-v\left(t\right)\right]e^{-kI^{2}\left(t\right)}+s\left(t\right) \end{array}$$

where *u(t), v(t), s(t)*, and *z(t)* are the state variables of excitatory, inhibitory, input, and output neurons, respectively; *f* is the sigmoid function; *a*_1_, *a*_2_, *b*_1_, and *b*_2_ are the weight parameters for these constitutive neurons; θ_*u*_ and θ_*v*_ are the thresholds for excitatory and inhibitory neurons; *I(t)* is the external input stimulus; and k is the decay constant.

**Figure 1:**
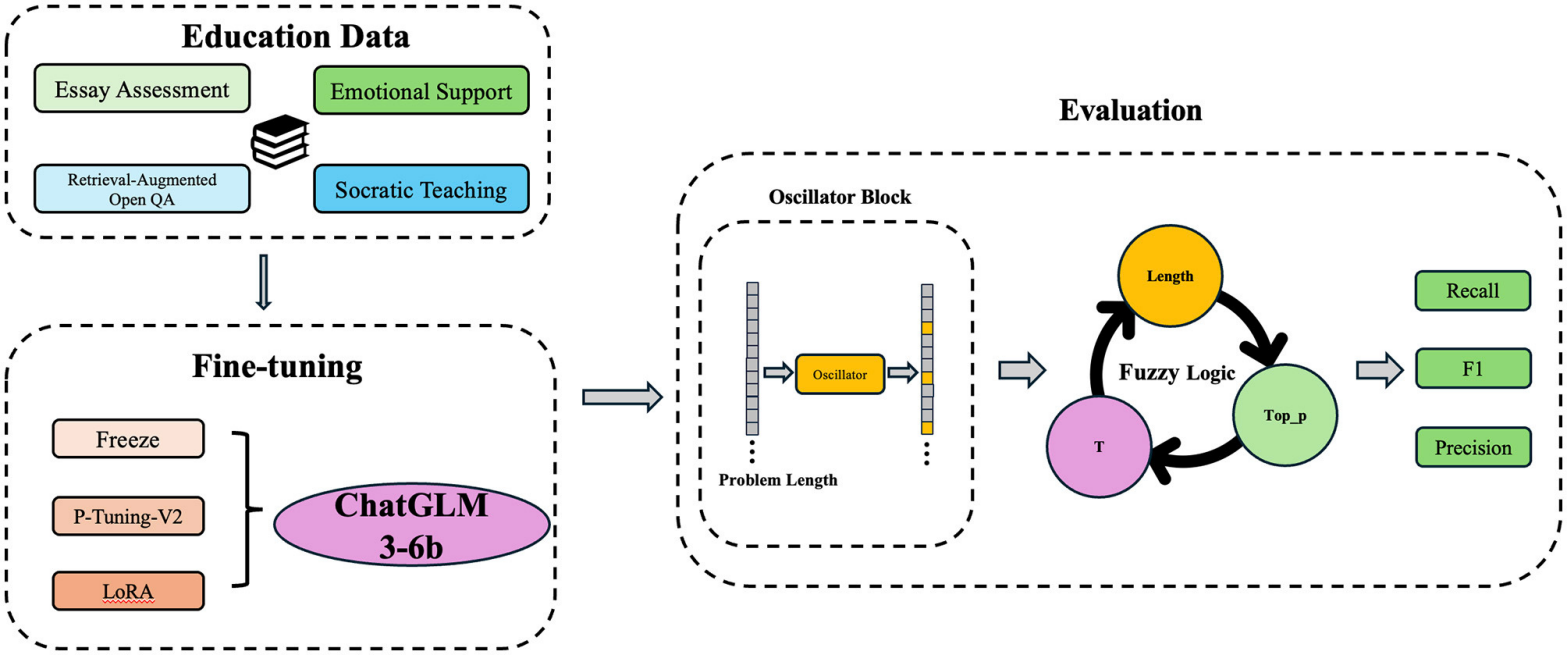
Framework of Chaotic LLM-based Educational Q&A System (CHAQS).

Hence, this study uses ChatGLM3-6b as a baseline model and integrates a Chaotic LLM-based Educational Q&A System to dynamically modify model parameters. It aims to refine response quality and contextual relevance generated by educational Q&A systems to address the specific needs of students and educators with flexibility and precision. Figure 1 shows the overall framework of CHAQS.

## Methodology

### Proposed LLM-based educational Q&A system (CHAQS)

This section uses different fine-tuning techniques, such as P-tuning v2, LoRA, and freeze, to fine-tune the baseline model ChatGM3-6b prior to Fuzzy Logic and Lee Oscillator integration.

P-tuning v2 fine-tuning technique is built upon P-tuning, to introduce dynamic prompt generation using a neural network to create context-aware prompts based on the input text. This allows flexible and effective task adaptation.

LoRA proposes an alternative adaptation strategy by introducing low-rank matrices that modify the self-attention and feed-forward layers of transformer models. This allows selective and efficient parameter updates to enhance model performance with minimal computational cost.

LoRA's modification of a transformer layer's weight matrix *W'* is described in Equation 5:


(5) $$\begin{array}{l} W^{{\;}^{\prime }}=W+B \end{array}$$

where *W* is the original weight matrix and *B* is the low-rank matrix learned during training. This approach provides a balance between adaptation flexibility and computational efficiency.

Freeze fine-tuning technique allows the remaining unfrozen parts to adapt to the specifics of a new task. It balances between frozen and trainable parameters and assists in maintaining the model's robustness while ensuring task-specific adaptability.

Hence, the baseline model ChatGLM3-6b uses the specific educational dataset by the above fine-tuning techniques prior to Fuzzy Logic and Lee Oscillator integration.

### Chaotic Fuzzy Logic Augmented Q&A System

**Figure 2:**
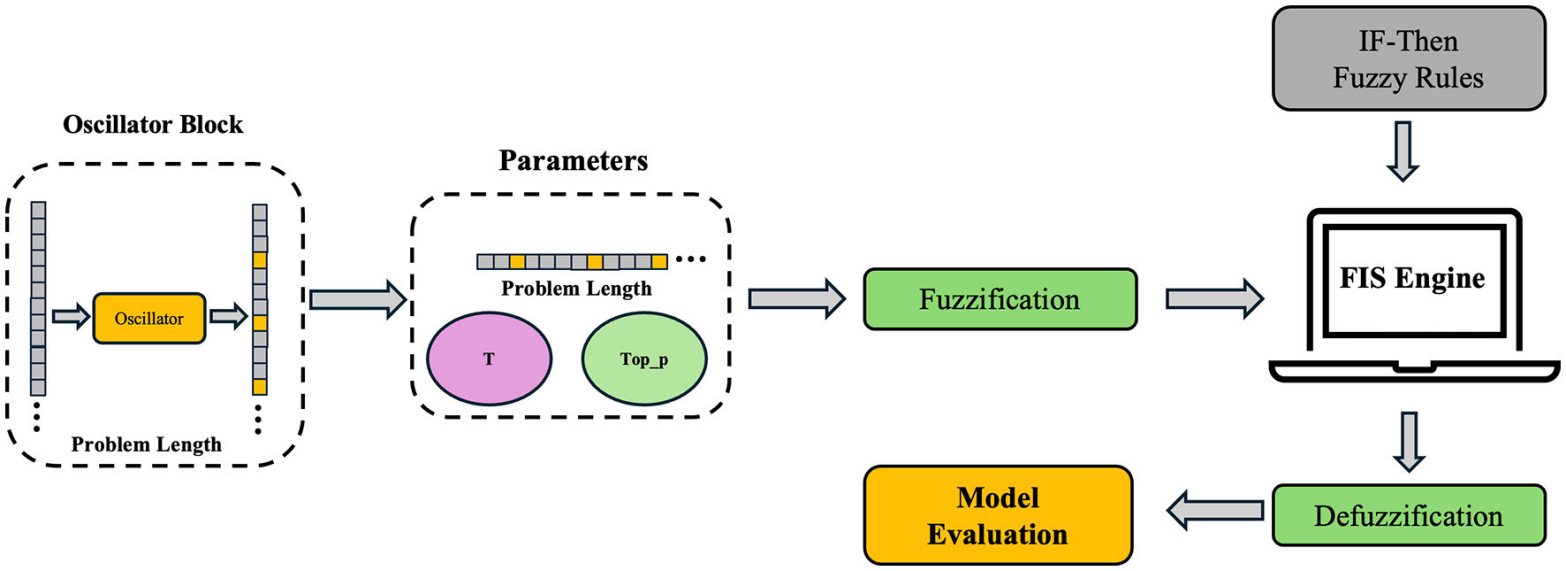
Structure of Chaotic Fuzzy Logic Augmented Q&A System.

Figure 2 shows a Chaotic LLM-based Educational Q&A System (CHAQS) structure. It consists of two parts, namely, Fuzzy Logic and Lee Oscillator, to optimize the answer generation process of the model after fine-tuning techniques are used to the baseline model ChatGLM3-6b. It also combines question texts to dynamically adjust model parameters, such as *top*_*p* and *temperature*. Chaotic Fuzzy Logic Augmented Q&A System procedure are listed in Algorithm 1.

**Algorithm 1:**
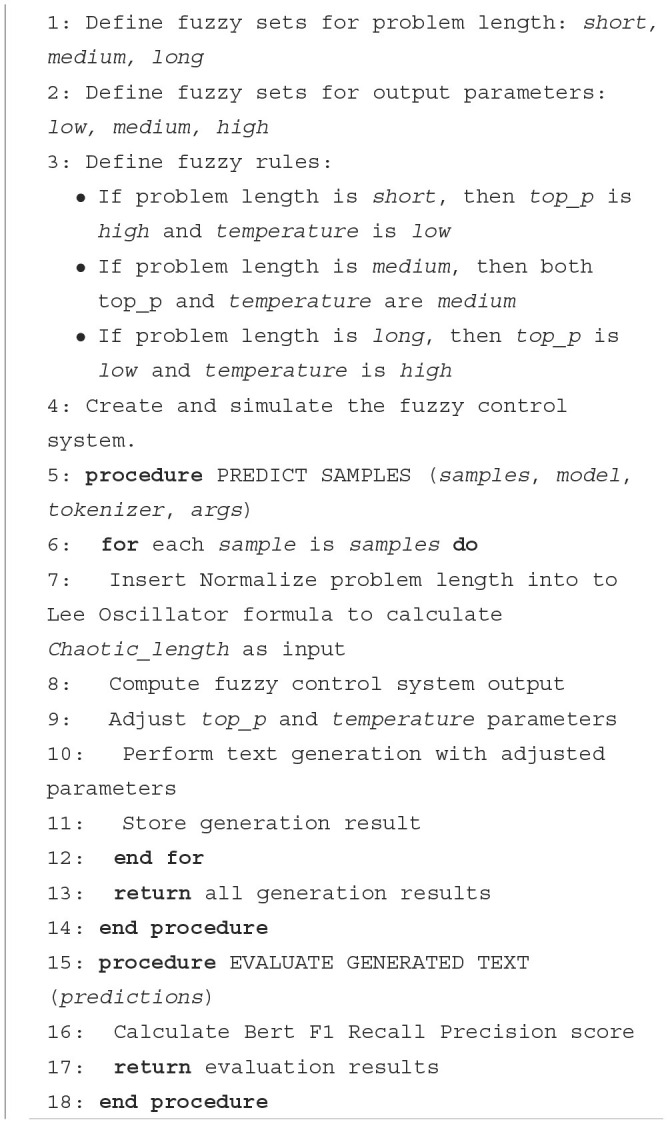
Chaotic Fuzzy Logic for adjusting educational Q&A system.

The *top*_*p* is a parameter of text generation strategy called “nucleus sampling,” and its basic idea is to randomly select the next word from a subset of vocabulary with the highest probabilities, where the cumulative probability of the subset reaches at least the threshold *p*. This approach aims to balance between diversity and coherence of the generated text by considering only the vocabulary whose cumulative probabilities reach a specific percentage, thereby avoiding an over-representation of high-frequency words while reducing the likelihood of generating irrelevant or illogical text. Moreover, the *top*_*p* mathematical representation is as follows: Let *V* be the vocabulary, for a given threshold *p*, find the smallest subset *V*′⊆*V* such that $\underset{w\in V^{\prime }}{\sum }P\left(w\right)\geq p$, where *P*(*w*) is the model output probability for word *w*. When generating the next word, sampling is performed from the set *V*′ according to its probability distribution.

The *Temperature* parameter is different from the parameter top_p. This parameter is used to control the randomness and creativity in the generated text by LLMs, adjusting the probabilities of predicted words in the output layer of the system. The original probability distribution calculated by the softmax layer is given as follows in Equation 6:


(6) $$\begin{array}{l} p\left(X_{i}\right)=2\times \frac{e^{X_{i}}}{\overset{V}{\underset{j=1}{\sum }}e^{X_{j}}}, \end{array}$$

where *X*_*i*_ is the logit corresponding to the i-th element and *V* is the total number of elements. Moreover, *p*(*X*_*i*_) is the resulting probability of the i-th element after applying the softmax function.

With the introduction of the *Temperature* parameter (*T*), the formula becomes Equation 7:


(7) $$\begin{array}{l} p\left(X_{i}\right)=2\times \frac{e^{\frac{X_{i}}{T}}}{\overset{V}{\underset{j=1}{\sum }}e^{\frac{X_{j}}{T}}}. \end{array}$$

To incorporate Fuzzy Logic into the system, the first step involves fuzzification of variables. Here, the length of the question text in the dataset as a variable is selected to define fuzzy membership functions accordingly. The set of fuzzymembership functions is as follows in Equation 8:


(8) $$\text{problem}\_\text{length}(x)=\{\begin{array}{ll} 1 & \text{if }0\leq x\leq 0.35\text{ (short)}\\ \frac{x-0.35}{0.5-0.35} & \text{if }0.35<x<0.5\text{ (medium)}\\ \frac{0.65-x}{0.65-0.5} & \text{if }0.5\leq x<0.65\text{ (medium)}\\ 1 & \text{if }0.65\leq x\leq 1\text{ (long)}\\ 0 & \text{otherwise} \end{array}$$

**Figure 3:**
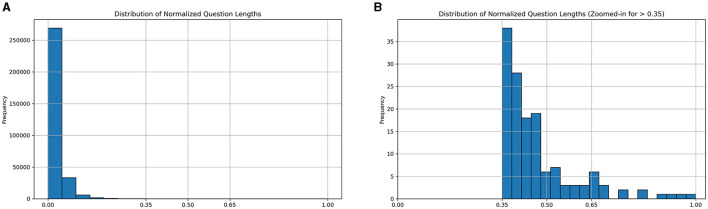
Histograms of the distributions of normalized question lengths. **(A)** Distribution of normalized question lengths. **(B)** Distribution of normalized question lengths (Zoomed-in for >0.35).

Referring to the case study of Fuzzy Logic Introduction (Lee, 2019), we classified the question length into three types: short, medium, and long. After that, as shown in Figure 3, we analyzed and visualized the question length distribution of this educational dataset. During the preliminary analysis, we observed that some significant breakpoints could be set as thresholds. For example, we identified that setting the thresholds at 0.35 and 0.65 effectively classified examples in this dataset, ensuring that our system can distinctly differentiate between the categories, thereby enhancing the ability of the model to generate contextually appropriate responses.

This categorization is crucial for the Fuzzy Logic system, allowing for a fuzzification process of problem length beyond mere numerical values. This fuzzy membership function enables the system to handle the inherently fuzzy nature of problem length classification, where the boundaries between categories are not rigid but gradual.

After defining the problem_length function, the *top_p* and *temperature* parameters generated by the model are fuzzified. These two parameters are categorized into the following terms: low, medium, and high, and their membership functions are defined as follows in Equations 9 and 10:


(9) $$\text{top}\_\text{p}(x)=\{\begin{array}{ll} 1 & \text{if }0.7\leq x\leq 0.8\text{ (low)}\\ \frac{x-0.85}{0.9-0.85} & \text{if }0.85<x<0.9\text{ (medium)}\\ \frac{0.87-x}{0.87-0.85} & \text{if }0.8\leq x<0.85\text{ (medium)}\\ 1 & \text{if }0.9\leq x\leq 1\text{ (high)}\\ 0 & \text{otherwise} \end{array}$$

(10) $$\text{temperature}(x)=\{\begin{array}{ll} 1 & \text{if }0.7\leq x\leq 0.8\text{ (low)}\\ \frac{x-0.85}{0.9-0.85} & \text{if }0.85<x<0.9\text{ (medium)}\\ \frac{0.87-x}{0.87-0.85} & \text{if }0.8\leq x<0.85\text{ (medium)}\\ 1 & \text{if }0.9\leq x\leq 1\text{ (high)}\\ 0 & \text{otherwise} \end{array}$$

Here, Lee Oscillator is integrated to dynamically modify the problem length parameters as follows (Equation 11):


(11) $$\begin{array}{l} Chaotic\text{\_}Problem\text{\_}length=\;Lee\left(Problem\text{\_}length\right) \end{array}$$

The calculation formula of the Lee Oscillator function *Lee*() is stated in Equations 1–4.

**Figure 4:**
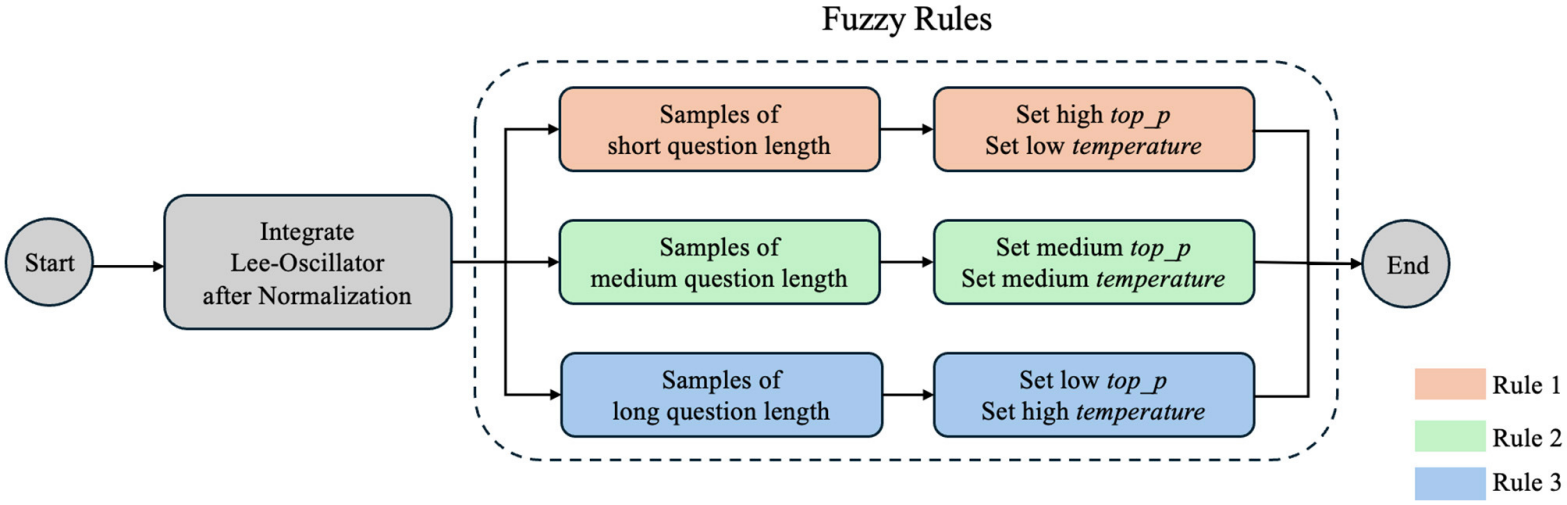
Fuzzy rules setting of CHAQS.

It is necessary to establish fuzzy rules for the system upon Lee Oscillator implementation to process the problem length. The length of the problem is used as a feature to adjust the *top_p* and *temperature* parameters of the system in its output answers, making diversified responses across different question lengths. Figure 4 shows the fuzzy rules setting of our system.

These Fuzzy Logic rules allow for varying levels of diversity and randomness in responses based on the length of the question.


(12) $$\begin{array}{l} Rule\;1:{Chaotic\text{\_}Problem\text{\_}length}_{high}\to \;\left({top\text{\_}p}_{high},temperature_{low}\right) \end{array}$$

As for short questions (Rule 1), a lack of information is often encountered. To introduce a suitable level of diversity in generated answers, the *top_p* parameter is set to a high range, allowing the model to explore a broader vocabulary space. At the same time, the *temperature* parameter is set to a low range to maintain the coherence of answers to reduce randomness, the Rule 1 is defined in Equation 12.


(13) $$\begin{array}{l} Rule\;2:{Chaotic\text{\_}Problem\text{\_}length}_{medium}\to \\ \left({top\text{\_}p}_{medium},temperature_{medium}\right) \end{array}$$

For medium-length questions (Rule 2), they generally provide sufficient background information so that the system does not require a high degree of creativity to provide reasonable answers. Therefore, the *top_p* parameter is set to a medium range, encouraging the model to focus on vocabulary and concepts already mentioned in the question text. However, to prevent the generated answers from being monotonous, the *temperature* parameter is set to a medium range to introduce a certain degree of innovation the Rule 2 is defined in Equation 13.


(14) $$\begin{array}{l} Rule\;3:{Chaotic\text{\_}Problem\text{\_}length}_{low}\to \\ \left({top\text{\_}p}_{low},temperature_{high}\right) \end{array}$$

For longer questions (Rule 3), since the question itself usually provides sufficient information, the *top_p* value is set to a low range to limit the vocabulary space of the model when predicting answers, encouraging it to focus on words closely related to the question text; at the same time, to avoid generating trivial or repetitive content, the *temperature* parameter is set to a high range to introduce creativity and unexpected elements in answers the Rule 3 is defined in Equation 14.

## Experiments

### Data description

The dataset used in the experiments is an English data version compiled by Dan et al. (2023). It contains numerous instructions and dialogue data, discussions related to role-playing, creative writing, and code-related topics and is organized by “problem” and “solution” fields to define the problem and resolution for each educational sample, as shown in Table 1.

**Table 1:** Subset of the dataset samples.

**Problem**	**Solution**
Rewrite the sentence \“He is running\” in the present progressive tense.	He is currently running.
Please put the following sentence in the correct order: I go swimming often times.	I often go swimming.
Generate two adjectives related to the following noun Caterpillar	Furry, wriggling.
Classify the following emails as \“SPAM\” or \“NON-SPAM\”: Hello! I am writing to offer you a free trial of my new product.	SPAM
Classify the following vegetables into their corresponding families: carrot, broccoli, onion, tomato, asparagus, and kale.	Carrot: Apiaceae \nBroccoli: Brassicaceae \nOnion: Amaryllidaceae\nTomato: Solanaceae\nAsparagus: Asparagaceae\nKale: Brassicaceae

**Figure 5:**
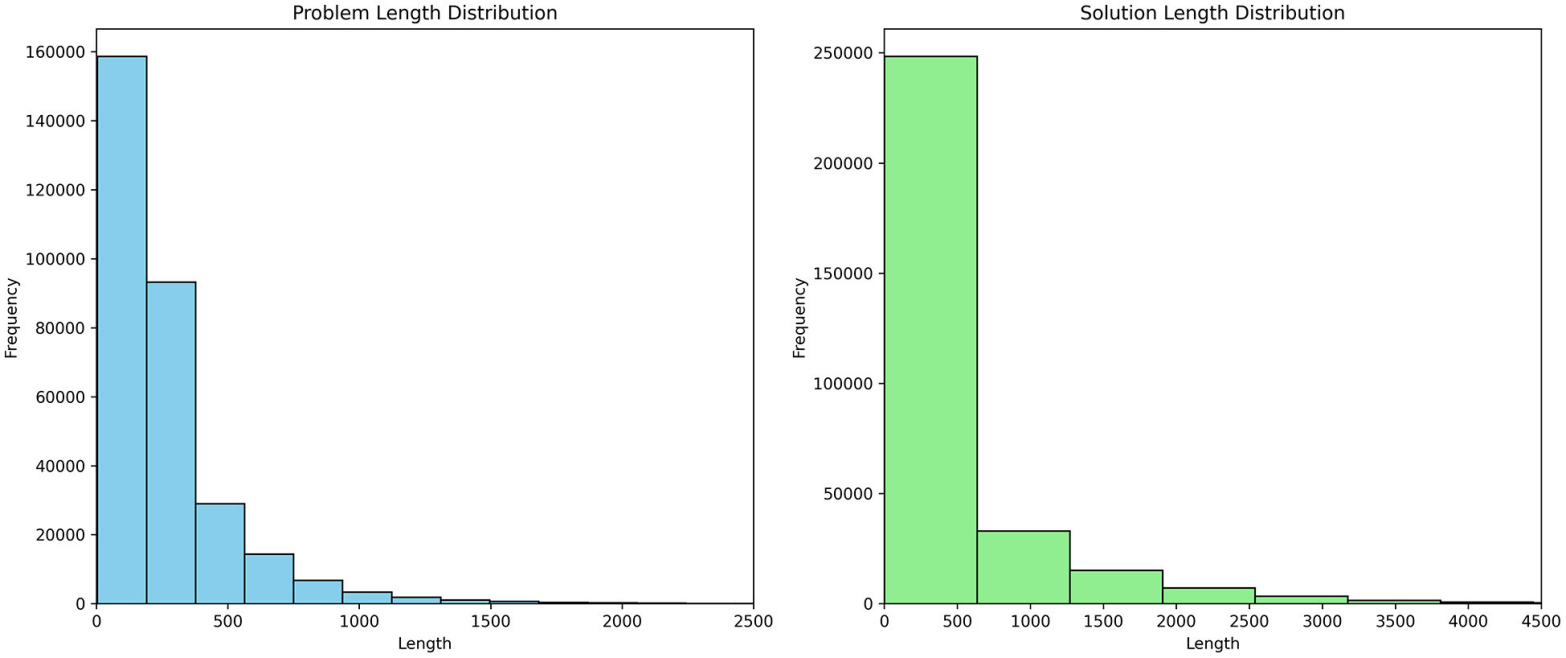
Histograms of the distributions of dataset example length.

Figure 5 shows histograms of dataset example length distributions. It is instrumental in determining the optimal maximum sequence length parameter for subsequent fine-tuning of the baseline model ChatGLM3-6b. It can identify a suitable parameter to accommodate the majority of data by examining sample length distribution in the educational dataset, ensuring that the baseline model ChatGLM3-6b is effectively fine-tuned on representative samples to avoid unneeded computational costs from excessive long sequences.

### Evaluation metrics

#### BERT score

We use BERT F1, precision, and recall score as evaluation metrics. BERT score (Zhang et al., 2019) is a metric to evaluate output quality in text generation tasks, such as machine translation or text summarization. It calculates the similarity between the predicted text and the reference text and uses a pre-trained BERT model (or other similar transformer models) to extract text embeddings and compute similarity scores accordingly. BERT score consists of three main steps: embedding, similarity calculation, and aggregation, which is calculated as follows:

- Embedding: The process is to translate words from reference texts, which are often referred to as standard texts, and the candidate texts are referred to as model answer texts into high-dimensional vector space *E*_*r*_ and *E*_*c*_, respectively.
- Similarity Calculation: The similarity between each pair of words is calculated in the reference text for the candidate text. Cosine similarity is used to calculate between *E*_*r*_ and *E*_*c*_ as shown in Equation 15:

(15) $$\begin{array}{c} similarity\left(E_{r},E_{c}\right)=\frac{E_{r}\;E_{c}}{\left\|E_{r}\right\|\;\left\|E_{c}\right\|} \end{array}$$

- Aggregation: The similarity score for the entire text is calculated. For *Precision* (*P*), the score for each candidate word *W*_*c*_ is the score of its highest similarity with words *W*_*r*_ in the reference text, as described in Equation 16. For *Recall* (*R*), the score for each reference word *W*_*r*_ is the score of its highest similarity with words *W*_*c*_ in the candidate text, as shown in Equation 17. Then, these scores are averaged to calculate the overall precision and recall, where the F1 score is the harmonic mean of precision and recall, as defined in Equation 18.

(16) $$\begin{array}{c} Precision=\frac{1}{\left|C\right|}\sum_{W_{c}\in C}\max_{r\in R}similarity\left(E_{r},E_{c}\right) \end{array}$$

(17) $$\begin{array}{c} Recall=\frac{1}{\left|R\right|}\sum_{W_{r}\in R}\max_{c\in C}similarity\left(E_{r},E_{c}\right) \end{array}$$

(18) $$\begin{array}{c} F_{1}=2\times \frac{P\times R}{P+R} \end{array}$$

#### Bilingual evaluation understudy

To verify the effectiveness of our system from multiple aspects, we also use bilingual evaluation understudy (BLEU; Papineni et al., 2002), to evaluate the quality of answers. It measures the similarity between the predicted text and the reference text by comparing n-grams. BLEU consists of three main steps: n-gram extraction, precision calculation, brevity penalty, and aggregation, which is calculated as follows:

- 1.N-Gram Extraction: N-gram extraction extracting n-grams from both the reference texts and the candidate texts.
- Precision Calculation: The precision of n-grams of the candidate text is calculated compared to the reference text. The precision for each n-gram length n is given by Equation 19.

(19) $$\begin{array}{l} P_{n}=\frac{\underset{C\in Candidates}{\sum }\underset{n-gram\in C}{\sum }\min\left(Count_{clip}\left(n-gram\right),Count_{ref}\left(n-gram\right)\right)}{\underset{C\in Candidates}{\sum }\underset{n-gram\in C}{\sum }Count\left(n-gram\right)} \end{array}$$

*Count*_*clip*_(*n*−*gram*) is the clipped count of a particular n-gram in the candidate translation. *Count*_*ref*_(*n*−*gram*) is the maximum count of a particular n-gram in the reference translation. *Count*(*n*−*gram*) is the total count of the n-gram in the candidate generation without clipping.

- Brevity penalty (BP): This process is designed to avoid favoring shorter generations. The BP calculation is defined in Equation 20.

(20) $$\begin{array}{c} BP=\{\begin{array}{c} 1,\;if\;c>r\\ e^{1-\frac{r}{c}},\;if\;c\leq r \end{array} \end{array}$$

where c is the length of the candidate text and r is the length of the reference text.

- Aggregation: The final BLEU score is computed in Equation 21.

(21) $$\begin{array}{c} BLEU\;=\;BP\;\cdot \exp(\sum_{n=1}^{N}w_{n}\log P_{n}) \end{array}$$

where **w**_**n**_ is the weight for n-grams.

#### Human evaluation

We also choose a human evaluation approach to evaluate our system comprehensively. A total of 10 evaluators participated in this part, reviewing 100 educational answers for each model. A previous study on the evaluation of LLM generation (Shen et al., 2023; Evans et al., 2024; Yang et al., 2024) considers various factors, such as coherence, consistency, fluency, and relevance. As for our educational domains, mathematical computing and problem-solving tasks are also included in the dataset. To assess the ability of the system in these tasks, we added an additional factor, accuracy, so our human evaluation includes five factors:

- Coherence: Apply coherence to evaluate whether the contents flow logically.
- Consistency: Evaluate whether the answer remains as contradictory statements.
- Fluency: Assess the readability of the answer.
- Relevance: Determines the relevance between the answer and the reference text.
- Accuracy: Checks the accuracy of the answer in some specific mathematical questions.

Each factor scores on a scale of 0–2, where 0 represents the answer is bad or false, 2 represents a suitable and accurate answer, and 1 represents an unclear partially incorrect answer. Therefore, each sample can achieve a maximum score of 10. The final human evaluation scores in the experimental table are calculated. First, the scores for all five factors are summarized for each sample. Then, the average of these total scores for all samples is computed, and this averaged score represents the final evaluation for each model.

### Baseline model

ChatGLM3-6b (Du et al., 2022) is selected as the open-source baseline model and fine-tuned with the dataset using techniques such as Freeze P-tuning-v2 (Liu et al., 2022b), and LoRA (Hu et al., 2022). CHAQS is used for the output of the model to provide quality responses.

### Experimental settings and hyperparameters

The hardware implementation is based on eight NVIDIA V100 Tensor Core GPUs, each with a 32 GB VRAM Graphics Card, using deep learning framework PyTorch 1.13.1+cu117, numpy 1.21.6, and pandas 1.3.5 for model fine-tuning. The specific hyperparameter settings of the experiment are shown in Table 2.

**Table 2:** Hyperparameter settings.

**Parameters**	**Parameters size**
Max length	1,560
Max source length	1,024
Learning rate	1.00E-04
Weight decay	0.1
Epochs	10
Training set proportion	0.8
Early stopping	3
Length of tunable token	16
LoRA rank size	16
LoRA alpha	64

### Model comparison

BERT F1, recall, precision scores, BLEU, and human evaluation are selected as evaluation metrics for the experiment. Here, Lee Oscillator parameter bifurcation behavior under different external input stimuli is used to capture the remarkable feature of the oscillator (Lee, 2004), and the specific oscillator parameter settings are presented in Table 3.

**Table 3:** Oscillator parameter settings.

**Parameter**	**Value**
Decay constant k	500
	5
	5
	1
	1

Using ChatGLM3-6b as the baseline model with different fine-tuning techniques on educational data, LoRA, Freeze, and P-tuning-v2 are used as stated previously. Subsequently, chaotic characteristics are introduced into various fine-tuned models by incorporating Fuzzy Logic and Lee Oscillator. The comparison of fine-tuning techniques is presented in Table 4.

**Table 4:** Model comparison with fine-tuning techniques.

**Technique**	**Model**	**Precision**	**Recall**	**F1**	**BLEU**	**Human**
ChatGLM3-6b		87.26	85.69	86.40	10.04	6.29
Freeze	Freeze-Fuzzy Logic	89.29	90.44	89.82	22.44	8.10
	Freeze-Chaotic Fuzzy Logic	89.68	91.26	90.42	21.42	8.06
LoRA	LoRA-Fuzzy Logic	90.46	91.32	90.84	23.15	7.78
	LoRA-Chaotic Fuzzy Logic	90.88	91.93	91.36	23.23	8.26
P-tuning-v2	P-tuning-v2-Fuzzy Logic	90.06	91.14	90.54	19.44	7.86
	P-tuning-v2-Chaotic Fuzzy Logic	90.75	92.07	91.36	20.84	8.12
	Imp.	4%	7%	6%	52%	23%

It showed that CHAQS has significant improvements across various metrics. It is evident that the fine-tuned model using the P-tuning-v2 technique has achieved BERT precision scores, recall scores, and F1 scores at 90.75, 92.07, and 91.36, respectively. It achieved improvements at 4, 7, and 6% compared to the baseline model, ChatGLM3-6b, which showed that it can enhance model performance, and our system also exhibited excellent performance according to both BLEU metric and human evaluation standards.

Moreover, CHAQS is compared with LLMs at a similar scale as a benchmark. These include (1) Qwen-7B-Chat (Bai et al., 2023), by Alibaba Cloud, is a model with 7 billion parameters, which enhances performance based on the Qwen-7B using an alignment mechanism; (2) Baichuan2-7B-Chat (Yang et al., 2023), by Baichuan Intelligence, is trained on 2.6 trillion tokens of high-quality corpus with commendable results on multiple authoritative English and multilingual general and domain benchmarks; (3) Llama-2-7B-Chat (Touvron et al., 2023), based on the Llama architecture with 7 billion parameters, is a model that has fine-tuned with human instructions based on the Llama-2-7B; (4) XVERSE-7B-Chat, by Shenzhen Yuanxiang Technology, is a mainstream decoder-only standard transformer network structure with a wide range of applications; (5) Yi-6B-Chat model, by (AI et al., 2024) 01.AI, is one of the open-source LLMs using 6 billion parameters and trained on 3 trillion bytes multilingual corpus; and (6) BlueLM-7B-Chat (Tack et al., 2023), by Vivo AI Global Research Institute, is a large-scale pre-trained language model that exhibits strong competitiveness among open-source models of similar scale.

It showed that CHAQS has significant improvements over other state-of-the-art models on the dataset in terms of BERT precision, recall, and F1 scores, as shown in Table 5. This highlights its effectiveness in this educational dataset. It also showed that there is an average increase of 5.12% in the precision score, an average increase of 11% in the recall metric, and an improvement of 8% in the F1 score compared with other models, and our system also exhibited excellent performance according to both BLEU metrics and human evaluation.

**Table 5:** State-of-the-art model comparison.

**Model**	**Precision**	**Recall**	**F1**	**BLEU**	**Human**
ChatGLM3-6b	87.26	85.69	86.40	10.04	6.29
Qwen-7B-Chat	86.70	81.58	84.00	7.43	5.93
Baichuan2-7B-Chat	86.56	81.88	84.07	9.09	6.46
Llama-2-7B-Chat	86.46	81.93	84.07	5.58	5.55
XVERSE-7B-Chat	86.18	82.65	84.30	3.18	4.04
Yi-6B-Chat	86.85	82.07	84.34	7.21	5.64
BlueLM-7B-Chat	85.23	84.14	85.55	13.94	6.60
Chaotic LLMs-based Educational Q&A System	90.75	92.07	91.36	20.84	8.12

### Ablation study

**Figure 6:**
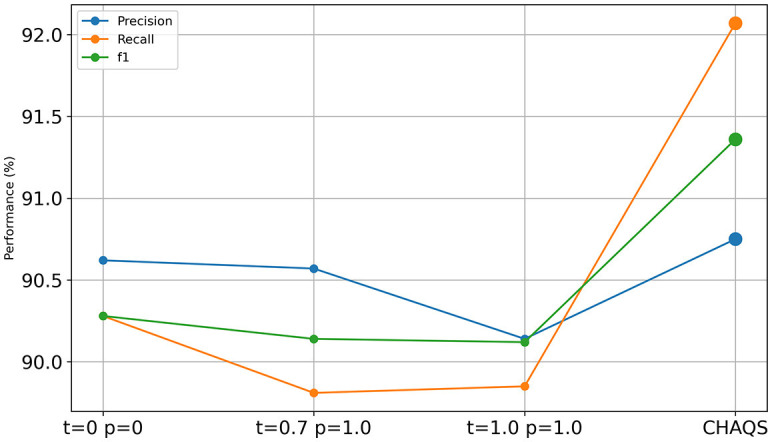
Ablation study of P-tuning v2 CHAQS.

**Figure 7:**
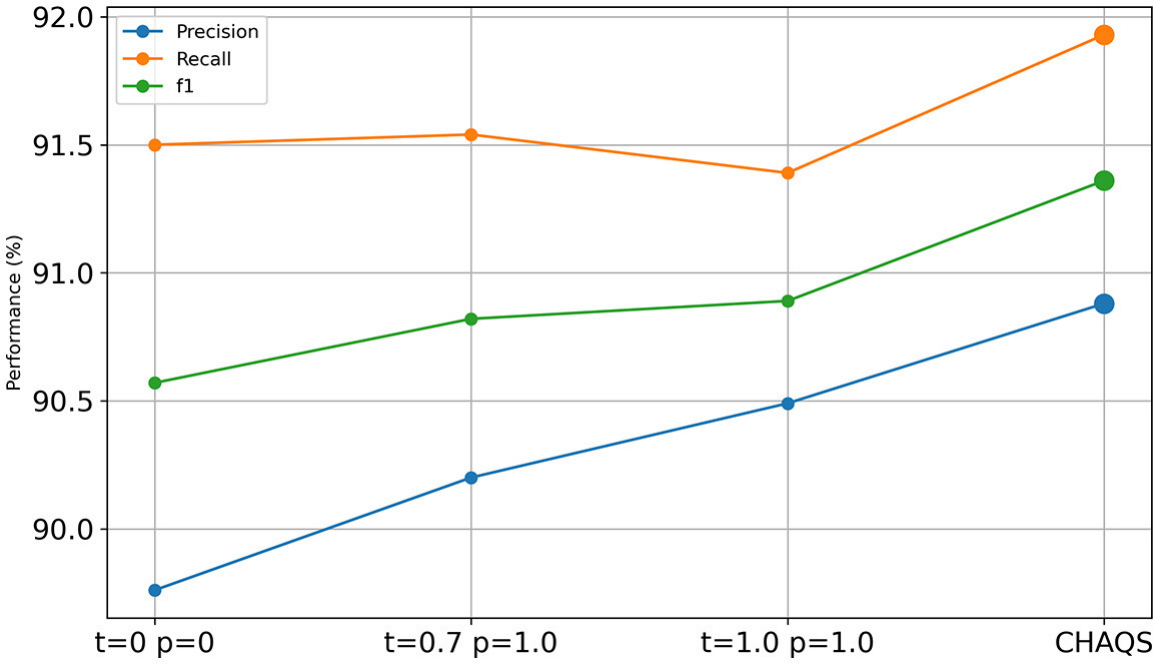
Ablation study of LoRA CHAQS.

Figures 6, 7 show the ablation study result of CHAQS. To highlight the impact of our method, we also compared it with other settings after P-tuning V2 and LoRA. We used different parameter settings, such as both *temperature* and *top_p (p)* set to 0 or 1, and referred to the OpenAI API (Goel et al., 2021), setting the *temperature* to 0.7 and the *top_p* to 1.0.

In addition, we also apply our CHAQS to llama2-7b-Chat (Touvron et al., 2023) using LoRA fine-tuning techniques, to validate the generality of our method, as shown in Table 6.

**Table 6:** A comparison between llama2-7b-chat and chamglm3-6b.

**Model**	**Technique**	**Precision**	**Recall**	**F1**	**BLEU**	**Human**
LLama2-7b-Chat		86.46	81.93	84.07	5.58	5.55
	LoRA-Fuzzy Logic	86.66	87.11	86.81	11.60	6.75
	LoRA-Chaotic Fuzzy Logic	87.21	87.87	87.49	18.33	7.86
ChatGLM3-6b		87.26	85.69	86.40	10.04	6.29
	LoRA-Fuzzy Logic	90.46	91.32	90.84	23.15	7.78
	LoRA-Chaotic Fuzzy Logic	90.88	91.93	91.36	23.23	8.26

### Case studies

There are three representative tasks selected to evaluate the performance and quality of the system: Task 1 determines relevance to a target, Task 2 converts given text into lemma form called lemmatization, and Task 3 analyzes a given text with candidate choices to select the most appropriate emotion. The comparison between CHAQS and ChatGLM3-6b answers from the test dataset is presented in Table 7.

**Table 7:** Model generation comparison.

**Problem**	**ChatGLM3-6b**	**CHAQS**
In this task, you are given two sentences and a target. You need to determine whether the first sentence is about the target or not. The second sentence will be about the target only if it immediately follows after a comma (,) or exclamation point (!). \nTarget: canaries \nCanaries were bred in captivity starting in the seventeenth century. They became popular pets because they did not require expensive cages like parrots. The output should be one of “yes” and “no.”	No	Yes
You are given an input text. Your task is to lemmatize the text and output its lemma form.\nNasser\u2019s regime change in Egypt. The output should contain only lowercase letters and spaces between words	Nass-'ers reg-i-me in e-gypt	Nassers\nregime\nchange\negypt
You are given a paragraph. Your task is to determine the emotion conveyed by the speaker in the paragraph. This can be one of five emotions—anger, anticipation, disgust, fear, or sadness. \nI had looked forward to my trip for months, and now it was finally here! Tomorrow morning I would board a plane and fly to New York City for 2 weeks of non-stop fun. The output should be one of five emotions—“anger,” “anticipation,” “disgust,” “fear,” or “sadness.”	Anticipate	Anticipation

It showed that CHAQS provided more accurate and task-appropriate answers. For instance, in Task 1, it is considered that the first sentence was related to the target “Canaries” accurately. In Task 2, CHAQS performed text lemmatization accurately and avoided incorrect hyphens and retention of separators. In Task 3, CHAQS also identified the emotion and selected the lemma form “anticipation” instead of “anticipate” accurately.

To highlight the impact of our method, we also compared it with other settings. For example, in Table 8, we used different parameter settings, such as both *temperature* and *top_p* (p) set to 0 or 1, and referred to the OpenAI API (Goel et al., 2021), setting the temperature to 0.7 and the *top_p* to 1.0.

**Table 8:** A comparison of different parameter settings.

**Problem 4**	**“Form a complex sentence with the given phrase. She buys clothes”**
*t* = 0 *p* = 0	She buys clothes.
*t* = 0.7 *p* = 1	She buys clothes for herself.
*t* = 1 *p* = 1	She buys clothes for herself.
Ours	She bought new clothes.
Problem 5	What emotion leads to punching?\\nOptions:\\n- fists\\n- hitting\\n- boxing gloves\\n- anger\\n- hands\nreasoning:
*t* = 0 *p* = 0	“Anger is an intense emotion. Anger leads to punching. The answer is anger.”
*t* = 0.7 *p* = 1	“Anger is an emotion. Anger leads to punching. The answer is anger.”
*t* = 1 *p* = 1	“Anger is an intense feeling of hostility or a strong feeling of badness. Anger leads to punching. The answer is anger.”
Ours	“Anger is a strong feeling of annoyance, displeasure, or hostility. Anger leads to punching. The answer is anger.”
Problem 6	A gentleman driving a blue car with the number 27 on it in race gear.\”\\nHypothesis: \”The man is winning the race.\”\\nIs the hypothesis entailed by the premise?\\nOptions:\\n- yes\\n- it is not possible to tell\\n- no.”
*t* = 0 *p* = 0	A person in a race is not necessarily winning the race. The answer: it is not possible to tell.
*t* = 0.7 *p* = 1	A man driving a car does not imply that the man is winning the race. The answer: it is not possible to tell.
*t* = 1 *p* = 1	A person can be in a race even if they are not winning. The answer: it is not possible to tell.
Ours	The car with the number 27 on it does not imply that the man is winning the race. The answer: it is not possible to tell.

As shown in Table 8, we selected representative questions to illustrate the diversity of responses in the system. For example, Problem 4 required generating a more complex sentence, and our CHAQS system produced the only sentence in the past tense. Regarding Problem 5, our CHAQS system provided a more detailed answer, not only offering a clear and concise definition of anger but also specifying its nature (annoyance, displeasure, and hostility). For Problem 6, which involves natural language inference to determine the relationship between a given premise and a hypothesis, our system not only provided the correct answer but also explained the process more clearly and accurately by pointing out that the car with the number 27 is unrelated to the problem requirements.

## Conclusion and future works

This study is focused on developing CHAQS that integrates with the LLM ChatGLM3-6b after fine-tuning with educational data. The approach is diverged from depending on commercial models and prompt-engineering practices via API calls opting for open-source models. Experiment results showed that CHAQS can significantly enhance the performance of LLM by using various fine-tuning techniques, integrating Fuzzy Logic and oscillators in the educational domain. When compared with various benchmark models, such as ChatGLM3-6b and Llama-2-7B-Chat, the system resulted in significant improvements in BERT precision, recall, and F1 scores, highlighting its effectiveness and potential for educational applications.

There are three representative tasks selected, namely, target relevance, lemmatization, and emotion analysis. It also showed that the system provided accurate answers compared with the benchmark model.

Nevertheless, there are limitations in the system. Fine-tuning parameters are required to improve response quality and coherence. Since the system is restricted by computational resources, it can only fine-tune small-scale open-source LLMs and is required to perform at other large-scale LLMs that have more than 10 billion parameters in various domains and tasks for evaluation. Hence, future studies can explore multi-turn Q&A, integrate OCR functions to process image-based educational data, and evaluate other approaches, such as assessments by experts or LLMs.

## Data availability statement

The raw data supporting the conclusions of this article will be made available by the authors, without undue reservation.

## Author contributions

HC: Conceptualization, Formal analysis, Investigation, Methodology, Project administration, Software, Writing – original draft. NS: Data curation, Validation, Visualization, Writing – original draft. LC: Funding acquisition, Project administration, Writing – review & editing. RL: Funding acquisition, Resources, Writing – review & editing.
